# Tibial Spine Avulsion Injuries in Children and Adolescents: A Narrative Review of Anatomy, Management Strategies, and Return-to-Sport Considerations

**DOI:** 10.3390/healthcare14131967

**Published:** 2026-07-02

**Authors:** Demah M. Benfaris, Zyad A. Aldosari, Abdulaziz S. AlNahari, Mohannad W. Awwad, Mohammed N. Alhuqbani, Abdulaziz Z. Alomar

**Affiliations:** 1 Department of Orthopedics, College of Medicine, King Saud University Medical City, King Saud University, Riyadh 11451, Saudi Arabia; 2 College of Medicine, King Saud University, Riyadh 11451, Saudi Arabia

**Keywords:** tibial eminence fractures, pediatric knee, skeletally immature, anterior cruciate ligament, arthroscopic fixation, physeal preservation, rehabilitation, return to sport

## Abstract

Tibial spine avulsion injuries represent a distinct pattern of anterior cruciate ligament (ACL) injury in children and adolescents, reflecting the unique anatomical and biomechanical properties of the skeletally immature knee. Unlike midsubstance ACL ruptures, these injuries involve avulsion of the tibial insertion and pose specific diagnostic and therapeutic challenges. Management strategies remain heterogeneous, particularly for partially displaced fractures, with variation in surgical indications, fixation techniques, and rehabilitation protocols. This narrative review provides a structured synthesis of current evidence on the anatomy, biomechanics, clinical presentation, and management of pediatric tibial spine avulsion injuries. Nonoperative and operative approaches are compared, with attention to fixation strategies, complications, physeal considerations, and rehabilitation principles. Return-to-sport (RTS) outcomes are examined, with available evidence suggesting that RTS rates may be comparable between operative and nonoperative management in selected patients, although interpretation is limited by heterogeneous and predominantly retrospective data. Early mobilization appears important for reducing arthrofibrosis risk, while rehabilitation should be individualized. RTS decision-making remains inconsistent, with commonly used criteria largely extrapolated from ACL reconstruction literature and lacking validation in pediatric populations. Multifactorial assessment incorporating functional testing and patient-reported outcomes is increasingly advocated, although evidence for psychological readiness remains limited. Overall, the current literature is characterized by methodological heterogeneity and limited comparative data, restricting definitive conclusions. This review provides a clinically oriented synthesis while highlighting key uncertainties and priorities for future research.

## 1. Introduction

Anterior cruciate ligament (ACL) injuries in children and adolescents represent a growing clinical challenge. This increase is driven by rising sports participation. It is further influenced by unique injury patterns related to skeletal immaturity and the high functional demands of young athletes. Over the past two decades, the incidence of ACL injuries in patients aged 6–18 years has increased substantially, with a reported annual rise of 2.3% and an average incidence of 121 cases per 100,000 individuals per year [1]. This increase is particularly notable among female athletes, who demonstrate higher injury rates across most age groups, with peak incidence during adolescence [1].

**Figure 1:**
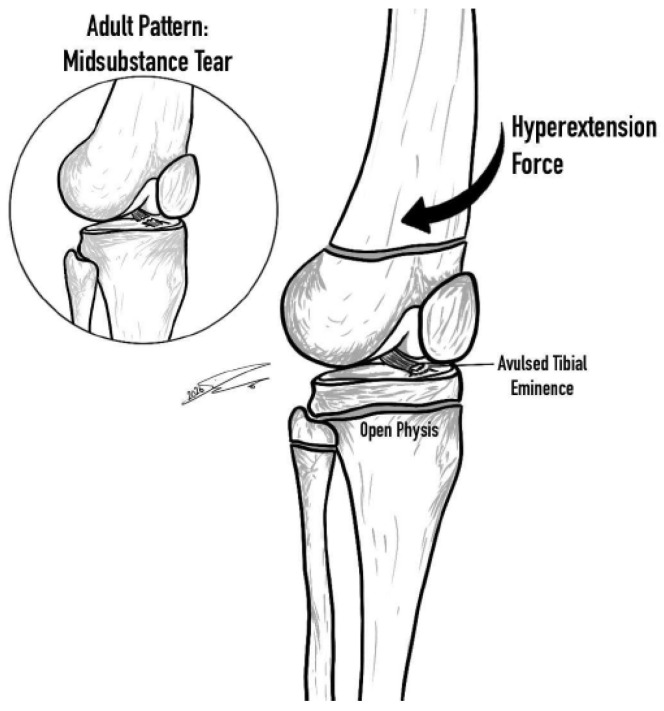
Schematic illustration of anterior cruciate ligament (ACL) injury patterns in skeletally immature and mature patients. Pediatric injuries typically involve avulsion of the tibial eminence with preserved ACL fibers, whereas midsubstance ligament rupture is more common after skeletal maturity. Original illustration created by a co-author.

In skeletally immature patients, ACL injuries frequently present as tibial spine (eminence) avulsion fractures rather than midsubstance ligament tears, as illustrated in (Figure 1). This pattern reflects failure of the incompletely ossified tibial eminence under tensile loading, while the ligament remains relatively stronger. Although injury mechanisms such as hyperextension, valgus loading, and rotational forces are shared with midsubstance ACL injuries, the resulting pathology differs due to developmental anatomy [2]. Tensile loading in the skeletally immature knee preferentially leads to avulsion of the tibial eminence rather than ligament rupture, reflecting the relative strength of the ACL compared to the incompletely ossified bone.

Anatomical factors appear to play a dominant role, as patients sustaining midsubstance ACL tears have narrower intercondylar notch dimensions than those sustaining avulsion fractures, independent of skeletal maturation status [2]. Tibial spine avulsion injuries most commonly occur between ages 8 and 14 years, account for 2–5% of pediatric knee injuries, and represent approximately 14% of all ACL injuries, with an incidence of 3 per 100,000 children annually [3]. This injury pattern reflects the evolving biomechanical relationship between ligament strength and bone integrity, with a gradual transition toward midsubstance tears as skeletal maturity is reached [3].

Despite overall favorable outcomes, the management of pediatric tibial spine avulsion fractures remains controversial, particularly for partially displaced (Meyers–McKeever type II) injuries [4]. While nondisplaced fractures are typically managed nonoperatively and completely displaced injuries are treated surgically, considerable variability persists in the management of partially displaced fractures, including differences in surgical indications, fixation strategies, and postoperative rehabilitation protocols [5]. Over time, there has been a shift toward operative management for type II and III fractures. This approach aims to restore ACL stability, address soft tissue interposition—most notably meniscal entrapment, reported in up to 40% of cases—achieve anatomic reduction, and reduce immobilization-related complications such as arthrofibrosis [5]. However, the predominance of retrospective and heterogeneous evidence limits the ability to define optimal management strategies, resulting in variability in clinical decision-making [4,5].

Return to sport has emerged as a critical outcome measure in pediatric knee ligament injuries, yet remains insufficiently standardized [6]. Although strength symmetry and functional testing are increasingly emphasized, RTS decision-making is often based on limited criteria, with nearly half of pediatric studies relying on a single assessment measure [7]. While high rates of return to sport have been reported in pediatric ACL populations, these data are largely derived from reconstruction cohorts and may not directly reflect outcomes following tibial spine avulsion injuries. Furthermore, factors contributing to reinjury risk are multifactorial, including biological healing, rehabilitation adequacy, and external pressures, and cannot be attributed solely to the absence of standardized RTS criteria. Psychological readiness is increasingly recognized as relevant to RTS, although evidence specific to tibial spine avulsion injuries remains limited, and commonly used metrics such as the Limb Symmetry Index continue to be debated in pediatric populations [7].

The existing literature on tibial spine avulsion injuries is further limited by predominantly retrospective study designs, small sample sizes, heterogeneous outcome measures, and inconsistent reporting of functional and sport-related outcomes [8]. In particular, evidence regarding rehabilitation protocols and RTS criteria remains sparse, with wide variability in reported timelines and a lack of validated pediatric-specific outcome measures [9,10]. These limitations highlight the need for a structured synthesis of current evidence.

Therefore, this narrative review aims to provide a comprehensive synthesis of current concepts related to the anatomy, biomechanics, evaluation, and management of tibial spine avulsion injuries in children and adolescents. Emphasis is placed on integrating available evidence on treatment strategies, rehabilitation considerations, and return-to-sport outcomes, with the goal of clarifying areas of consensus and ongoing uncertainty rather than establishing definitive recommendations.

## 2. Methods

This narrative review was conducted in accordance with the Scale for the Assessment of Narrative Review Articles (SANRA) guidelines to ensure methodological rigor, transparency, and balanced synthesis of the available literature [10].

Relevant literature was identified through structured searches of the PubMed and Google Scholar databases from January 2000 to January 2025. In PubMed, both free-text keywords and Medical Subject Headings (MeSH) were used where applicable, and search terms were combined using Boolean operators (AND/OR). Search terms included combinations of keywords related to the topic of interest, including “tibial spine avulsion,” “ACL avulsion,” “pediatric anterior cruciate ligament,” “adolescent ACL injury,” “tibial eminence fracture,” “surgical fixation,” “nonoperative management,” “rehabilitation,” and “return to sport.” A representative search strategy is provided in Supplementary Material File S1 to enhance reproducibility. Additional articles were identified through manual screening of reference lists from key publications to ensure comprehensive coverage of foundational and contemporary studies. The number of studies identified through manual screening was limited and served as a complementary search method. Preference was given to primary studies when supporting specific epidemiological, clinical, and outcome-related statements.

Studies were considered eligible if they addressed clinical, biomechanical, or outcome-related aspects of tibial spine avulsion injuries in pediatric or adolescent populations. Both operative and nonoperative management studies were included. Exclusion criteria comprised studies focusing exclusively on adult populations, non-English publications, and reports lacking sufficient methodological detail or clinical relevance.

To minimize potential selection and confirmation bias, literature screening and data extraction were performed independently by multiple authors. Discrepancies were resolved through discussion and consensus among the reviewing authors. The review included relevant clinical, biomechanical, and outcome-based studies involving pediatric and adolescent populations, encompassing both operative and nonoperative management strategies. Studies of varying design, including retrospective cohorts and comparative studies, were incorporated, with interpretation guided by their methodological strengths and limitations. Studies presenting supportive as well as conflicting findings were deliberately incorporated to provide a balanced and critical overview of current concepts.

Given the narrative design, formal risk-of-bias assessment tools and quantitative synthesis (e.g., meta-analysis) were not applied. Instead, evidence was interpreted qualitatively, with emphasis on study design, consistency of findings across studies, and clinical applicability. This approach was intentionally selected to allow synthesis of heterogeneous literature and discussion of evolving management strategies, rehabilitation principles, and return-to-sport considerations. While systematic reviews and meta-analyses exist on selected aspects of tibial spine avulsion injuries, the scope of this review was broader, integrating anatomical, biomechanical, clinical, and rehabilitative domains that are not consistently addressed within a single systematic framework.

## 3. Results and Discussion

The following sections present a clinically oriented synthesis of current evidence, organized thematically from anatomical and epidemiological foundations through to management strategies, rehabilitation, and return-to-sport considerations.

### 3.1. Anatomy and Biomechanics

The anterior cruciate ligament (ACL) in skeletally immature patients exhibits distinct anatomical and developmental characteristics compared with the mature knee, influencing both injury patterns and management strategies [11]. The femoral origin of the ACL is located eccentrically on the lateral femoral condyle, closer to the deep articular cartilage, and, in most knees, has an oval insertional footprint with substantial individual variation in its dimensions and area [12]. Importantly, the femoral ACL origin remains entirely epiphyseal throughout development and is consistently positioned approximately 3 mm distal to the distal femoral physis from mid-gestation through adolescence, without significant migration during growth [11].

The distal femoral physis is the most active growth plate in the human body, contributing approximately 1 cm of longitudinal growth annually and accounting for 70% of femoral and 37% of total lower limb growth, while the proximal tibial physis contributes approximately 0.7 cm per year, representing 55% of tibial and 25% of lower limb growth [11]. The tibial ACL insertion has a characteristic C-shaped footprint measuring 12.6 mm in width and 3.3 mm in thickness, with the anteromedial bundle positioned anterior and medial to the posterolateral bundle within the anterior intercondylar region [13].

During skeletal maturation, the angle between the ACL and the tibial plateau progressively increases in both sagittal and coronal planes, with younger children demonstrating significantly smaller angles that only reach adult-like orientation at skeletal maturity [11]. This evolving orientation presents a challenge in skeletally immature patients, as graft placement based on adult anatomy may result in excessive graft steepness and compromised rotational stability once growth is complete [11]. In addition, pediatric knees demonstrate greater ligamentous laxity than adult knees, while immature meniscal and cartilage tissues exhibit enhanced healing potential due to increased vascularity, greater thickness, and absence of subchondral ossification [11].

The distinct injury pattern observed in skeletally immature patients—tibial spine avulsion fractures rather than midsubstance ACL tears—is primarily related to the relative mechanical properties of bone and ligament during development. In this population, the ligamentous structures are relatively stronger than the incompletely ossified tibial eminence, predisposing the bone–ligament interface to failure under tensile loading [14,15]. Biomechanical studies have shown that loading characteristics play a critical role in determining the mode of failure. Lower loading rates tend to result in tibial eminence avulsion fractures, whereas higher loading rates are more likely to produce midsubstance ACL tears [15].

Furthermore, cadaveric analyses demonstrate that younger specimens exhibit higher biomechanical values yet fail predominantly through bony avulsion when tested in anatomical orientation, reflecting the relative strength of ligamentous structures compared with their osseous attachments [14]. It is also important to recognize that failure is not purely a binary process. Before complete bony failure, an in situ stretch injury of the ACL may occur, contributing to residual laxity even after successful fracture reduction and healing [15]. Morphometric analyses further support this mechanism, demonstrating wider tibial eminence dimensions in patients with avulsion fractures compared with those sustaining midsubstance ACL tears, suggesting that increased mediolateral dimensions of the tibial ACL footprint may predispose to bony failure under load [14].

### 3.2. Epidemiology and Mechanisms of Injury

Tibial spine avulsion fractures are relatively uncommon but clinically significant injuries in the pediatric population, with an estimated incidence of 3 per 100,000 children annually, accounting for 2–5% of pediatric knee injuries and approximately 14% of all ACL injuries across age groups [16]. The largest epidemiological analysis to date, involving 876 cases from a multi-institutional database, reported a male predominance with a male-to-female ratio of 2.2:1, although case distribution was nearly equal between sexes up to ages 9–10 years, after which injury rates diverged [16]. Peak incidence occurred at 13–14 years for boys and 11–12 years for girls, findings corroborated by a systematic review of 36 studies including 766 pediatric patients with a mean age of 12.4 years (range 3–18 years) [5,16].

The mechanisms underlying pediatric ACL avulsion injuries have shifted over recent decades, with sports-related activities now representing the most common etiology, replacing the historically predominant mechanism of bicycle falls [5]. A systematic analysis demonstrated that sports accounted for 36.2% of tibial spine avulsion injuries, followed by bicycle falls (21.1%), motor vehicle accidents (16.4%), skiing (14.5%), and other mechanisms (11.8%) [5]. Skiing has emerged as a particularly relevant mechanism in certain geographic regions, accounting for the majority of cases in some series [5]. Injury typically occurs through hyperextension combined with valgus or rotational forces, resulting in failure of the immature subchondral bone before rupture of the ACL substance, particularly during athletic activities involving sudden quadriceps contraction and high tensile stress on the patellar tendon–ACL complex [5].

### 3.3. Classification Systems

#### 3.3.1. Meyers and McKeever Classification of Tibial Spine Avulsion Injuries

**Figure 2:**
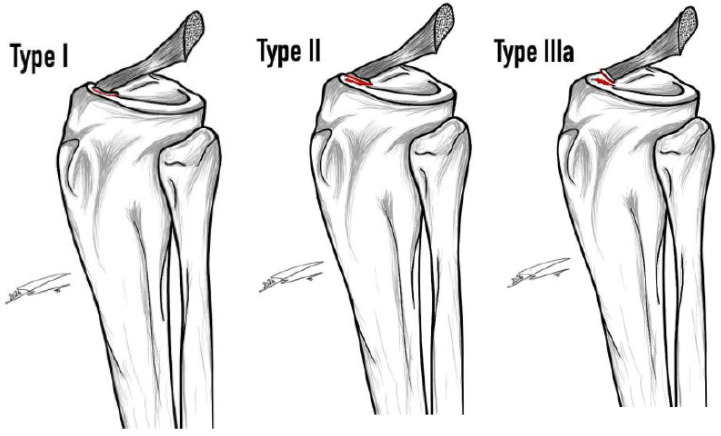

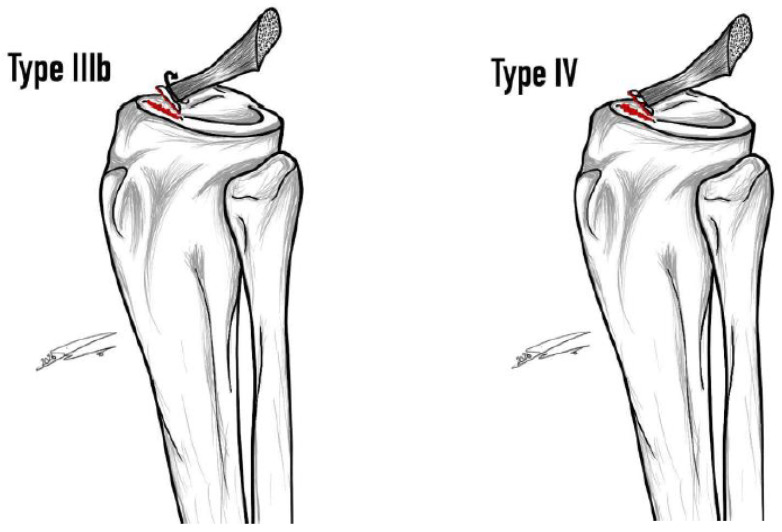
Modified Meyers–McKeever classification of tibial spine avulsion fractures (Zaricznyj modification), illustrating Type I (nondisplaced or minimally displaced), Type II (anterior elevation with intact posterior hinge), Type III (IIIa and IIIb) (complete displacement), and Type IV (comminuted or rotated fragment). Original illustration created by a co-author.

The classification system introduced by Meyers and McKeever for fractures of the intercondylar eminence of the tibia remains a widely used framework for describing tibial spine avulsion injuries in children, based on the degree of displacement of the avulsed fragment [17]. The original system defines three patterns: Type I injuries with minimal or no displacement and good apposition, Type II injuries with anterior elevation of the fragment and an intact posterior hinge, and Type III injuries with complete separation of the fragment from the tibial eminence [17,18]. Subsequent pediatric series have confirmed that the system reliably distinguishes nondisplaced Type I fractures from more displaced injuries, although differentiating between partially and completely displaced fractures (Types II and III) can be challenging on plain radiographs because the integrity of the posterior hinge is not always clearly visualized [18,19]. The spectrum of fracture displacement patterns described by the modified Meyers and McKeever classification is illustrated in Figure 2.

Clinically, this classification informs management strategies, with Type I fractures generally treated nonoperatively using cast or brace immobilization, Type II fractures often managed with closed or arthroscopic reduction and immobilization when an acceptable reduction is achieved, and completely displaced or comminuted fractures (Types III and IV) typically requiring operative reduction and fixation to restore ACL function and minimize residual laxity or loss of motion [17,18,19]. Despite its widespread use, the system shows only moderate inter- and intraobserver reliability, with reported kappa values ranging from 0.47 to 0.69 for radiographic and MRI assessment, underscoring variability in fracture grading among clinicians [20].

#### 3.3.2. Zaricznyj Modification and Clinical Application

Zaricznyj later modified the Meyers and McKeever classification by adding a Type IV category to describe comminuted tibial eminence avulsion fractures that are not adequately captured by the original three types [21]. These highly displaced or comminuted injuries are usually managed operatively because reliable closed reduction is rarely achievable and fragment size or rotation often necessitates suture-based or screw fixation techniques to obtain stable reduction [18,19].

With the increasing use of MRI and arthroscopy, several series have highlighted that tibial spine avulsion fractures are frequently associated with additional intra-articular pathology, including meniscal entrapment, meniscal tears, osteochondral injury, and other ligamentous lesions, which are not fully characterized by radiograph-based classification alone [19,22]. In a large multicenter cohort, meniscal entrapment was identified in approximately 40% of patients with tibial spine avulsions, and both MRI and surgical findings demonstrated substantial rates of concomitant meniscal, chondral, and non-ACL ligamentous injury, supporting the role of advanced imaging when planning treatment [22]. An MRI-based classification proposed by Green and colleagues demonstrated higher diagnostic accuracy (91.2–95.6%) compared with the Meyers and McKeever system (73.5–76.5%), and altered treatment recommendations in nearly one-third of cases by incorporating quantitative displacement and soft tissue assessment [20]. Nevertheless, the Meyers and McKeever classification, together with the Zaricznyi modification, remains the most commonly cited and clinically applied system [20].

### 3.4. Clinical Features of Tibial Spine Avulsion Injuries in Adolescents

Tibial spine avulsion fractures typically present with acute knee pain and swelling following trauma, most commonly during sports or falls involving hyperextension with valgus or rotational forces [8,23]. Physical examination usually reveals a tense hemarthrosis, moderate to severe effusion, restricted range of motion—particularly loss of terminal extension—and difficulty bearing weight [24]. Unlike midsubstance ACL tears in adults, patients frequently report a distinct “pop” at the time of injury, reflecting avulsion of the tibial spine by the ACL [23]. A positive Lachman test is commonly present, indicating anterior laxity, although assessment may be limited by pain and joint effusion [24]. Hemarthrosis is a hallmark feature and occurs more consistently than in isolated ligamentous injuries due to the bony nature of the lesion [25]. Careful evaluation for associated injuries is essential, as meniscal entrapment occurs in up to 40% of cases and concomitant ligamentous injuries may be present, particularly following high-energy trauma [23].

### 3.5. Role of Radiographs and MRI in Diagnosing Pediatric ACL Avulsion Injuries

Plain radiographs are the initial imaging modality and should include anteroposterior, lateral, and tibial tunnel views, with lateral radiographs best demonstrating fracture displacement [23]. Type II injuries often display the classic “bird’s beak” appearance due to anterior fragment displacement with an intact posterior hinge [8]. However, radiographs are limited in visualizing nonossified cartilaginous components and associated soft tissue injuries, especially in younger children [24]. Small avulsed fragments may appear subtle and risk being misinterpreted as incidental findings despite representing clinically significant pathology [24].

Magnetic resonance imaging (MRI) is therefore essential for comprehensive assessment, allowing evaluation of both osseous and soft tissue structures, confirmation of ACL fiber continuity, and identification of associated injuries that influence treatment planning [24]. In a retrospective study of 82 pediatric knees with ACL injuries, tibial spine avulsions were significantly more common in skeletally immature patients (26%) than in skeletally mature individuals (4%), with MRI frequently demonstrating bone contusions (79%), joint effusion (84%), anterior tibial translation (72%), and characteristic lateral compartment contusion patterns (71%) [25]. MRI reliably identifies meniscal entrapment, interstitial ACL injury, collateral ligament and posterolateral corner injuries, and meniscal tears, all of which may alter surgical decision-making and prognosis [23]. Diagnostic accuracy for complete ACL injury exceeds 90% in pediatric populations, although partial tears remain more challenging to detect, and the presence of hemarthrosis or lipohemarthrosis further supports the diagnosis of tibial spine avulsion injury [25].

#### Nonoperative and Operative Management Principles

**Figure 3:**
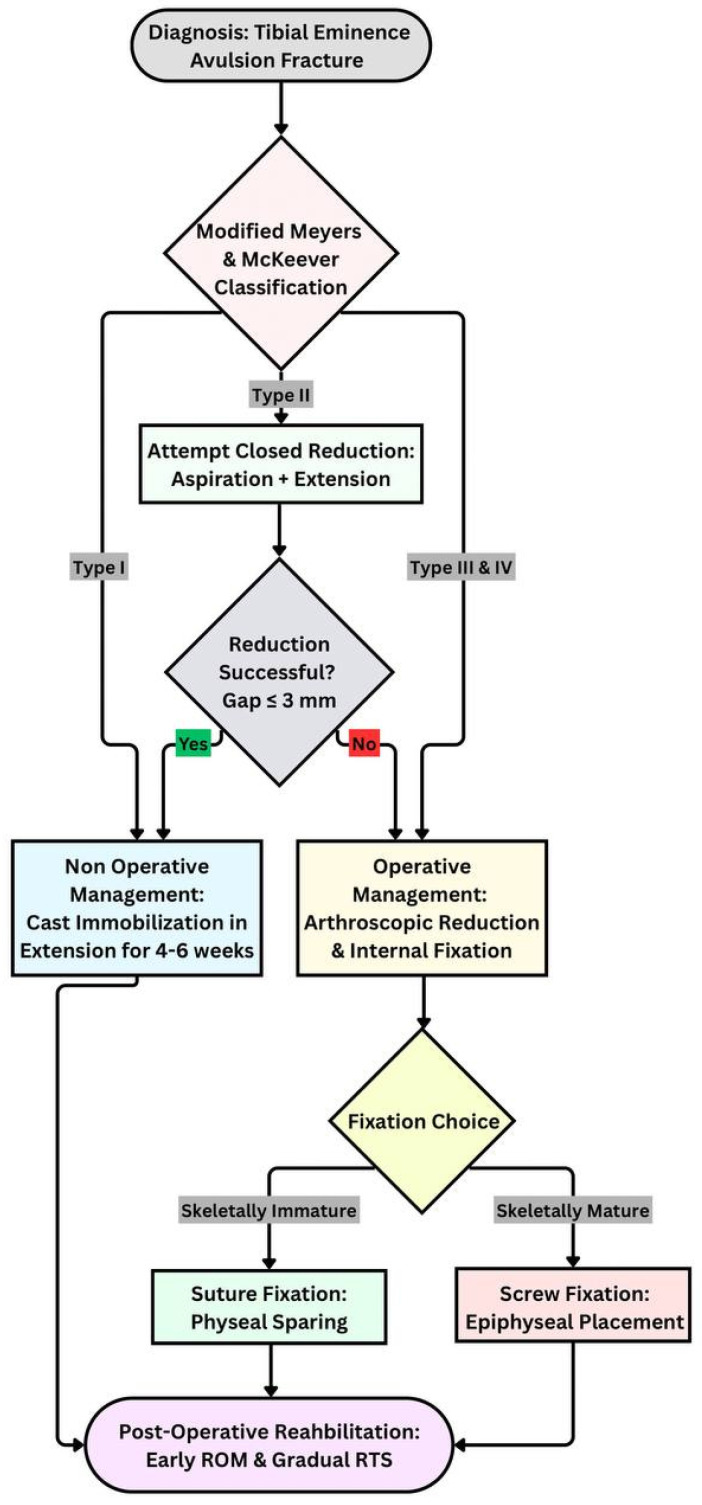
Management algorithm for pediatric tibial spine avulsion fractures based on fracture displacement, reducibility, skeletal maturity, and fixation strategy. Successful closed reduction is defined as a residual gap of ≤3 mm; failure to achieve this threshold is an indication for operative management. Original illustration created by a co-author.

A simplified treatment algorithm for pediatric tibial spine avulsion fractures, integrating fracture displacement, reducibility, and skeletal maturity, is shown in Figure 3.

## 4. Nonoperative Management

Nonoperative management remains an appropriate treatment option for selected pediatric patients with tibial spine avulsion injuries, particularly those with Type I fractures and carefully selected Type II injuries [15]. Conservative treatment typically consists of cast immobilization in extension for 4–6 weeks, with or without closed reduction and hemarthrosis aspiration [15]. Type I fractures generally demonstrate excellent healing with short-term immobilization, although minimizing immobilization duration is important to reduce the risk of knee stiffness and arthrofibrosis. [15].

Although overall complication rates following nonoperative treatment are comparable to operative management, differences in functional outcomes and residual laxity have been reported across studies [4]. A multicenter cohort study of 625 children treated nonoperatively found that 1.3% required subsequent ACL reconstruction and 2.6% were diagnosed with ACL insufficiency, with the highest risk observed in boys aged 13–16 years [4]. For Type II fractures, conservative management may be appropriate only when satisfactory closed reduction is achieved and maintained. However, failure of reduction is relatively common, often due to soft tissue interposition—most commonly involving the intermeniscal ligament or meniscus—which may necessitate conversion to operative management [15]. In such cases, MRI plays an important role in identifying intra-articular obstacles to reduction, which may be more clinically relevant than displacement measurements alone.

Residual anterior laxity represents the most frequently reported complication after nonoperative treatment and occurs at significantly higher rates than following operative fixation [4]. In comparative analyses of Type II fractures, 28% of nonoperatively treated patients demonstrated residual laxity on Lachman testing, compared with 7.2% in the operative group. However, the clinical significance of this finding remains variable, as a proportion of patients with objective laxity remain asymptomatic. Nonoperative management has also been associated with higher rates of subsequent ACL-related procedures in some cohorts, particularly in older adolescents [4,15]. These findings suggest that while nonoperative treatment is effective in selected cases, careful patient selection, radiographic assessment, and close clinical follow-up are essential to optimize outcomes.

## 5. Operative Management

Indications for surgical fixation of tibial spine avulsion fractures are primarily guided by the Meyers and McKeever classification system. There is consensus supporting nonoperative treatment for Type I fractures and operative intervention for Types III and IV, whereas the management of Type II fractures remains debated [26]. Surgical fixation is recommended for completely displaced (Type III) and comminuted or rotated (Type IV) fractures, as these patterns cannot be reliably reduced through closed methods and are associated with a high risk of persistent instability, nonunion, or malunion if left untreated [26].

Type II fractures represent the principal area of controversy. Operative management is generally indicated when closed reduction fails to achieve acceptable alignment, defined as residual displacement exceeding 3 mm (gap >3 mm), or when soft tissue interposition prevents anatomic reduction [26]. In this context, MRI may provide additional value by identifying intra-articular pathology, such as meniscal entrapment, which may influence treatment decisions beyond displacement measurements alone. Comparative studies suggest that surgical fixation of displaced fractures is associated with lower rates of residual instability and reduced need for subsequent ACL reconstruction; however, these findings are derived predominantly from retrospective and heterogeneous cohorts, limiting the strength of inference. [26].

Additional indications for arthroscopic reduction and internal fixation include concomitant intra-articular pathology, such as meniscal tears or entrapment, as well as associated ligamentous or chondral injuries requiring arthroscopic evaluation [25]. Early surgical intervention has been associated with improved outcomes in some studies, while delays and prolonged operative times have been linked to increased risk of arthrofibrosis [25]. Nevertheless, thresholds for timing and operative parameters vary across studies, and standardized recommendations are lacking. [26].

The primary goal of surgery is anatomic reduction of the avulsed fragment with restoration of ACL tension and knee stability, thereby preventing chronic instability, which has been reported in up to 70% of displaced fractures managed nonoperatively [8]. Secondary goals include removal of interposed soft tissue, elimination of mechanical blocks to extension, and stable fixation that allows early mobilization while preserving physeal integrity [8,26]. While operative management may offer advantages in selected displaced injuries, particularly with regard to stability, current evidence does not uniformly demonstrate superiority across all patient groups, reinforcing the importance of individualized decision-making.

### 5.1. Surgical Techniques and Fixation Methods

#### 5.1.1. Arthroscopic Surgical Techniques for Tibial Spine Avulsion Injuries in Adolescents

Arthroscopic reduction and internal fixation (ARIF) has become the preferred surgical approach for tibial spine avulsion injuries in children and adolescents, largely replacing open techniques due to its minimally invasive nature and favorable clinical outcomes [26]. Reported advantages include smaller incisions, reduced postoperative pain and infection risk, faster recovery, improved visualization of the fracture site, and the ability to simultaneously address associated intra-articular pathology [26].

The fundamental principle of ARIF is direct arthroscopic visualization, which facilitates accurate reduction of displaced fragments and identification and removal of interposed soft tissue—most commonly meniscal entrapment, reported in 40–68.8% of cases—prior to fixation [26]. The procedure typically involves diagnostic arthroscopy, evacuation of hemarthrosis, fracture reduction, and stabilization using transosseous suture techniques with absorbable sutures. This method allows fixation of both simple and comminuted fractures while avoiding routine secondary procedures for hardware removal [26]. Clinical outcomes following ARIF are generally favorable, with approximately 85% of pediatric patients achieving good to excellent International Knee Documentation Committee scores and most regaining satisfactory function within six months [26].

#### 5.1.2. Comparison of Screw Fixation and Suture Fixation for Pediatric Tibial Spine Fractures

**Figure 4:**
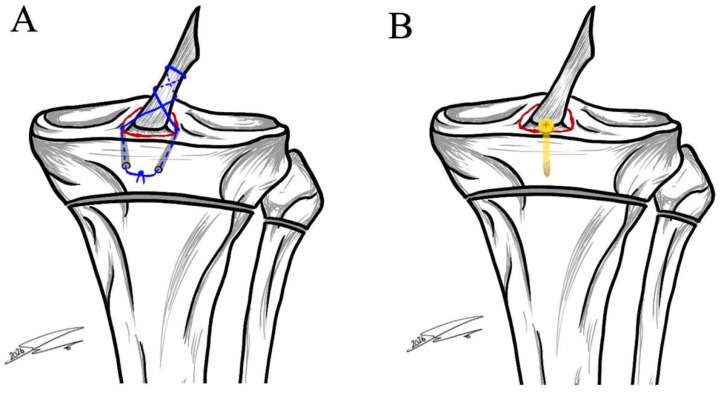
Common fixation techniques for tibial spine avulsion fractures: (**A**) physeal-sparing transosseous suture fixation and (**B**) screw fixation with epiphyseal placement. Original illustration created by a co-author.

Common fixation strategies for pediatric tibial spine avulsion fractures, including physeal-sparing suture constructs and screw fixation techniques, are illustrated in Figure 4.

Screw fixation and suture fixation remain the most commonly employed methods for stabilizing pediatric tibial spine avulsion fractures, each with distinct biomechanical and clinical profiles [27]. Biomechanical studies have yielded variable results depending on experimental models: adult and porcine studies suggested higher peak failure loads and improved cyclic stability with suture constructs, whereas pediatric cadaveric studies demonstrated no significant differences in ultimate load between double-screw and suture fixation, despite greater ligament stiffness observed with screws [27]. Importantly, pediatric models consistently demonstrate lower failure loads than adult specimens, limiting extrapolation of adult biomechanical data to skeletally immature patients [27].

Clinically, a comparative study of 68 children with Type II and III fractures found no significant differences between suture and screw fixation in rates of arthrofibrosis, postoperative instability, range of motion, ACL reconstruction, or overall return to sport [27]. However, screw fixation was associated with significantly higher reoperation rates, largely due to implant removal, with 65% of patients in the screw group requiring reoperation compared with 39% in the suture group (odds ratio 2.9; *p* = 0.03) [27]. Radiographic healing occurred sooner with suture fixation (3 vs. 5 months), although greater fragment elevation was observed, without adverse impact on clinical outcomes [27].

A systematic review of pediatric-only studies demonstrated superior functional outcomes with suture fixation, including higher Tegner, International Knee Documentation Committee, and Lysholm scores, as well as a significantly lower need for revision surgery, albeit with a higher reported incidence of arthrofibrosis [28]. Emerging fixation strategies—such as transosseous sutures, suture anchors, and double-row suture bridge constructs—have shown promising biomechanical properties, with double-row techniques demonstrating the highest failure loads and least displacement during cyclic testing [28]. A meta-analysis further identified higher risks of revision surgery and implant removal following screw fixation, particularly in patients with open physes, supporting the preferential use of physeal-sparing, suture-based techniques in skeletally immature patients [29].

### 5.2. Complications and Growth-Related Considerations

#### 5.2.1. Complications Following Surgical Fixation of Tibial Spine Avulsion Injuries in Children

Complications after surgical fixation of tibial spine avulsion injuries remain clinically relevant, with arthrofibrosis representing the most frequent and impactful postoperative complication [30]. In a multicenter cohort of 249 pediatric patients, arthrofibrosis occurred in 23.3%, defined by ≥10° extension loss and/or ≥25° flexion loss at three months or the need for manipulation under anesthesia and lysis of adhesions, with 7.6% ultimately requiring surgical intervention for stiffness [30]. A systematic review and meta-analysis of 38 studies including 1179 operatively treated children demonstrated significantly higher complication rates following screw fixation compared with suture fixation (13.0% vs. 2.9%; odds ratio 5.01; 95% CI, 2.0–12.4; *p* = 0.0006), encompassing postoperative instability, reoperation, and persistent pain [8].

Other reported complications include residual ACL laxity, which occurs in approximately 10% of operatively treated patients compared with 22% following nonoperative management, as well as anterior knee pain, motion loss, nonunion, malunion, and infection, although reported incidences vary across studies [30].

#### 5.2.2. Growth Disturbance and Physeal Safety After Pediatric ACL Avulsion Surgery

Growth disturbance is a recognized but incompletely defined risk following surgical treatment of pediatric ACL injuries, with risk varying according to surgical technique and physeal involvement [31]. A large systematic review of 2693 pediatric ACL reconstructions identified growth disturbances in 2.6% of patients, including angular deformities, limb length discrepancies, and altered tibial slope, with some patients exhibiting abnormalities in multiple planes [31]. Although tibial spine avulsion injuries involve fixation at the ACL insertion rather than ligament reconstruction, principles derived from pediatric ACL reconstruction literature remain applicable for physeal safety considerations [31].

Extraphyseal techniques are associated with the lowest risk of growth disturbance, whereas intraepiphyseal femoral tunneling carries a higher risk of angular deformity, particularly valgus malalignment [31]. Specific deformity patterns have been linked to distinct mechanisms, including eccentric physeal arrest (valgus), central arrest (shortening), intraepiphyseal tunneling (lengthening), and anterior tibial physeal injury (reduced tibial slope) [31]. Growth-related complications may be minimized by limiting physeal violation through drill diameters <6 mm, steep drilling angles, avoidance of the physeal periphery, and avoidance of hardware, bone plugs, or synthetic materials crossing the physis or leaving drill holes empty [31].

Notably, hardware or bone plugs traversing the physis have been strongly associated with localized growth arrest requiring corrective procedures, supporting preferential use of physeal-sparing suture fixation techniques over screw fixation in skeletally immature patients with tibial spine avulsion injuries [31]. While extraphyseal approaches demonstrate minimal growth disturbance, excessive graft tension should be avoided to prevent tenodesis effects, and although no graft ruptures were reported with allograft use in this review, allografts remain generally discouraged in pediatric populations for other clinical reasons [31].

### 5.3. Postoperative Rehabilitation

Postoperative rehabilitation following arthroscopic reduction and internal fixation of tibial spine avulsion fractures is essential to optimize recovery while protecting fracture healing and minimizing complications, particularly arthrofibrosis [26]. Rehabilitation is typically structured in progressive phases, beginning immediately after surgery with an early controlled range of motion limited to 0–90° during the first two weeks, which is intended to reduce stress on the fixation construct while minimizing the risk of arthrofibrosis associated with prolonged immobilization, followed by gradual advancement thereafter [26]. Partial weight-bearing with the knee brace locked in extension is commonly initiated around postoperative day three, progressing to full weight-bearing over four weeks, with discontinuation of crutches once tolerated [32]. Bracing is generally maintained for up to eight weeks to protect fixation. Early mobilization is emphasized given the markedly increased risk of arthrofibrosis when motion is delayed beyond four weeks [26]. However, it is important to note that these rehabilitation timelines are largely derived from small cohort studies and expert opinion, with limited high-level evidence supporting specific protocols, and the available literature on this topic remains concentrated among a limited number of primary sources. As such, variability in postoperative rehabilitation strategies remains common across institutions, and recommendations should be interpreted accordingly.

Rehabilitation following nonoperative management differs in that initial immobilization is often more prolonged, and progression of range of motion may be more cautious due to the absence of internal fixation, potentially delaying functional recovery compared with surgically treated patients. Early rehabilitation (weeks 0–6) focuses on edema control, pain management, protection of fixation, and prevention of stiffness, with passive range of motion progressing toward full flexion and initiation of isometric quadriceps and hamstring exercises [32]. Intermediate rehabilitation (weeks 7–12) advances strengthening, restores full motion, and incorporates proprioceptive and closed-chain exercises as the brace is gradually unlocked [32]. Advanced rehabilitation (weeks 13–18) introduces straight-line running after week 12, functional strengthening, agility drills, and neuromuscular training, followed by sport-specific conditioning and plyometrics between months 5 and 6 [32]. Return to daily activities is typically achieved by eight weeks, with non-contact sports permitted at three to four months and return to contact or competitive sports generally between four and six months, depending on individual progress and sport demands [26]. Return-to-sport decisions should not rely solely on time-based criteria or limb symmetry indices, as these may overestimate functional recovery. A multifactorial approach incorporating strength, neuromuscular control, and psychological readiness is recommended.

Rehabilitation following ACL avulsion repair differs from traditional ACL reconstruction, as restoration of the native ligament attachment preserves proprioceptive fibers and facilitates biological healing at the original insertion site. Patients undergoing tibial-sided ACL avulsion repair typically follow protocols that allow early controlled range of motion and progressive weight bearing within the first few postoperative weeks, supporting favorable early functional recovery [33]. Consequently, rehabilitation protocols after tibial spine fixation are often accelerated compared with graft-based reconstruction, while still emphasizing protection against reinjury and stiffness [26].

### 5.4. Return to Sport

Return to sport is a primary indicator of successful treatment following tibial spine avulsion injuries, although it should be interpreted alongside broader measures of recovery rather than as a sole indicator of treatment success. Most pediatric patients achieve functional recovery sufficient for athletic participation [34]. A prospective study of skeletally immature patients reported that 78% returned to their preinjury level of sport at long-term follow-up, with fear of reinjury—rather than pain or instability—being the most common reason for sports discontinuation among those who did not return [34]. Notably, available evidence suggests that RTS rates may be comparable between operative and nonoperative management in selected patient groups, although this observation is derived from limited and heterogeneous data. This highlights the potential importance of patient selection and rehabilitation strategies in influencing outcomes, rather than treatment modality alone [34].

Functional outcomes following tibial spine avulsion fixation are consistently excellent when assessed using pediatric-specific outcome measures. Long-term follow-up studies report high Pedi-IKDC scores (mean 96.4 ± 5.7) and favorable sports participation levels measured by the Pedi-FABS, with a substantial proportion of patients regularly engaging in pivoting and cutting activities [34]. Objective knee stability is restored in most cases, with a high proportion demonstrating normal anterior laxity on instrumented testing [34]. However, these findings should be interpreted with caution given the predominance of retrospective designs and variability in outcome reporting across studies.

These findings are supported by a systematic review of operatively treated pediatric patients with Meyers–McKeever Type II–IV fractures, which demonstrated excellent postoperative Tegner, IKDC, and Lysholm scores, reflecting high functional capacity and sports participation [35]. Among surgical techniques, suture fixation has been associated with comparable or, in some studies, improved functional outcomes relative to screw fixation, particularly in the context of physeal preservation [35]. Nevertheless, direct comparative evidence remains limited, and conclusions regarding superiority of specific fixation methods should be considered provisional. Overall, the literature suggests that many adolescents are able to return to sport following tibial spine avulsion injuries; however, variability in study design, patient selection, and outcome definitions limits the ability to draw definitive conclusions regarding optimal management strategies and long-term functional equivalence between treatment approaches. It should be noted that a proportion of the functional outcome data cited in this context is derived from broader pediatric ACL populations, including reconstruction cohorts, and may not fully reflect outcomes specific to tibial spine avulsion injuries [35].

### 5.5. Return-to-Sport Criteria and Decision Making

Return-to-sport (RTS) decision making in adolescent athletes after knee ligament injury requires a multifactorial approach, as no single metric reliably predicts safe return to activity [36]. Current evidence supports a multidimensional RTS assessment combining objective functional testing, psychological readiness, and clinical evaluation. Commonly recommended objective measures include strength testing with limb symmetry index (LSI) assessment of quadriceps and hamstrings (targets ≥ 90% symmetry or <10–20% deficit), balance assessment using the Y-Balance Test or single-leg stance, and hop testing (single-leg, triple, crossover, and 6 m timed hop tests) [37]. Thresholds such as ≥90% strength symmetry or <10–20% interlimb deficit are frequently cited; however, these values are largely extrapolated from ACL reconstruction literature and have not been specifically validated in pediatric tibial spine avulsion populations [37].

Psychological readiness is increasingly recognized as a relevant factor in RTS decision-making. Higher ACL–Return to Sport After Injury (ACL-RSI) scores have been associated with improved functional performance, while fear of reinjury and kinesiophobia remain important barriers to return [34,37]. However, evidence specifically evaluating psychological readiness in tibial spine avulsion injuries remains limited, and current understanding is largely derived from broader ACL injury populations.

Patient-reported outcome measures, including the International Knee Documentation Committee Subjective Knee Form and the Pediatric Functional Activity Brief Scale, may complement objective testing and provide additional insight into functional recovery [36]. Nevertheless, no standardized or validated RTS criteria currently exist for this patient population, and clinical decision-making remains largely individualized.

Taken together, RTS decision-making in adolescents with tibial spine avulsion injuries should be guided by a combination of functional recovery, patient-reported outcomes, and clinical judgment, while acknowledging the limitations of existing evidence and the lack of pediatric-specific validation of commonly used assessment tools.

### 5.6. Long-Term Outcomes and Prognosis

#### 5.6.1. Long-Term Knee Stability and Functional Outcomes

Long-term studies indicate that pediatric tibial spine avulsion injuries generally result in favorable knee stability and functional outcomes when appropriately managed, although residual anterior instability remains the most common long-term finding [34]. A multicenter cohort with mean follow-up of 87 months reported that 75.4% of patients returned to their preinjury level of sport, with a mean IKDC score of 90.9, reflecting good functional recovery [34]. At extended follow-up of up to 15.75 years, subjective outcomes and health-related quality of life remained high, with no significant differences between open osteosuturing and arthroscopic fixation techniques [38]. Nevertheless, 14.8% of patients returned to sport at a reduced level—primarily due to fear of reinjury, pain, or instability—and 9.8% were unable to return to sport [38]. These findings are consistent with other long-term series reporting stable knees and high RTS rates following appropriate treatment [34].

#### 5.6.2. Residual Instability and Risk of Osteoarthritis

Residual anterior instability is thought to result primarily from traumatic elongation of the ACL at the time of injury, rather than inadequate fracture fixation, and may persist despite anatomic reduction and fracture healing [38]. This in situ stretch injury has been observed across treatment modalities [38]. Concern also exists regarding long-term risk of posttraumatic osteoarthritis (PTOA), although data specific to tibial spine avulsion injuries remain limited. Evidence from pediatric ACL reconstruction provides indirect insight, with a large national study reporting a 5-year PTOA incidence of 1.6%, substantially lower than adult rates [39].

However, several factors significantly increased PTOA risk, including subsequent surgery for arthrofibrosis, age ≥12 years at injury, delayed surgical treatment ≥3 months, and obesity [39]. Notably, patients requiring lysis of adhesions or manipulation under anesthesia demonstrated a fivefold increased risk of PTOA [39]. Given that arthrofibrosis develops up to 12 times more frequently when postoperative mobilization is delayed beyond four weeks, timely diagnosis, appropriate surgical timing, and early rehabilitation are critical to minimizing both short-term complications and long-term degenerative sequelae in pediatric ACL avulsion injuries. It should be acknowledged, however, that the PTOA risk factor data cited above are derived from pediatric ACL reconstruction cohorts and have not been specifically validated in tibial spine avulsion populations, where dedicated long-term degenerative outcome studies remain lacking. [39,40].

### 5.7. Future Directions and Research Gaps

The current literature on pediatric and adolescent tibial spine avulsion injuries contains substantial gaps that limit the development of robust, evidence-based management guidelines [8]. A systematic review and meta-analysis of 38 studies involving 1237 patients emphasized the need for large-scale comparative research to clarify long-term outcomes associated with different fixation techniques [8]. Although arthroscopic reduction and internal fixation—particularly suture-based methods—is increasingly utilized, the available evidence remains predominantly retrospective, with heterogeneous methodologies and limited long-term follow-up, precluding definitive conclusions regarding optimal treatment approaches. These limitations hinder meaningful assessment of skeletal maturation effects, late functional sequelae, and long-term degenerative changes [8].

Future investigations should also focus on the clinical significance of concomitant intra-articular pathology, particularly meniscal entrapment and chondral injury, which are identified intraoperatively in approximately 40–68.8% of cases but are frequently underrecognized on preoperative magnetic resonance imaging [8]. Clarifying how these associated injuries influence treatment selection and outcomes may help refine surgical indications beyond fracture displacement alone [8].

A parallel and critical deficiency exists in the absence of validated, age-specific return-to-sport protocols for pediatric ACL-related injuries, including tibial spine avulsion fractures, which account for approximately 14% of cases [41]. An international Delphi consensus highlighted the need for multifactorial return-to-sport assessment incorporating clinical recovery, neuromuscular control, limb symmetry thresholds adjusted for pubertal status, and psychological readiness using instruments such as the Anterior Cruciate Ligament Return to Sport After Injury scale [41]. However, these recommendations are largely based on expert opinion and extrapolation from adult or ACL reconstruction populations, with limited validation in skeletally immature athletes [41].

Notably, the current literature suggests that return-to-sport rates may be comparable between operative and nonoperative management in selected patients; however, this observation has not been adequately explored in prospective or comparative studies. Further research is needed to determine whether treatment modality, rehabilitation strategies, or patient-specific factors primarily drive functional recovery and return to sport.

Consequently, future research should prioritize well-designed prospective and multicenter studies aimed at developing standardized, developmentally appropriate management algorithms and RTS criteria. Such efforts should integrate clinical outcomes, biomechanical considerations, and patient-reported measures to better inform individualized treatment strategies and long-term joint health in pediatric populations.

## 6. Conclusions

Tibial spine avulsion injuries represent a distinct pediatric manifestation of anterior cruciate ligament injury driven by the anatomical and biomechanical characteristics of the skeletally immature knee. Current management strategies are guided by fracture classification and clinical assessment; however, variability in treatment approaches persists, particularly for partially displaced injuries.

Available evidence suggests that both operative and nonoperative management can result in favorable functional outcomes in appropriately selected patients, although comparative data remain limited and heterogeneous. Arthroscopic reduction with physeal-sparing fixation is commonly utilized for displaced fractures, while nonoperative treatment remains appropriate in selected cases.

Return-to-sport decision-making should extend beyond time-based clearance and incorporate a multifactorial approach, including functional assessment and patient-reported outcomes. However, currently used criteria are largely extrapolated from ACL reconstruction literature and lack validation in pediatric tibial spine avulsion populations.

Although overall outcomes are generally favorable, the current evidence base is limited by retrospective study designs, heterogeneous outcome measures, and a lack of standardized protocols, which restricts the strength of clinical inferences.

Accordingly, management of these injuries should be individualized, with recognition of existing uncertainties. Future high-quality prospective studies are required to better define treatment indications, optimize rehabilitation strategies, and establish validated pediatric-specific return-to-sport frameworks.

## Data Availability

No new data were created or analyzed in this study.

## Supplementary Materials

The following supporting information can be downloaded at: https://www.mdpi.com/article/10.3390/healthcare14131967/s1, Supplementary Material File S1. Representative PubMed Search Strategy.
