# Supplementary material for: Tibial Spine Avulsion Injuries in Children and Adolescents: A Narrative Review of Anatomy, Management Strategies, and Return-to-Sport Considerations
Source: Healthcare (Basel). 2026 Jul 2;14(13):1967. doi: 10.3390/healthcare14131967 (PMC13361917; doi:10.3390/healthcare14131967)
Supplement: Supplementary file 1 [file healthcare-14-01967-s001.zip › healthcare-4260042-supplementary.pdf]

Supplementary Material File S1. Representative PubMed Search Strategy

A representative search strategy used for literature identification in PubMed is provided below to enhance transparency and reproducibility:

(“tibial spine avulsion” OR “tibial eminence fracture” OR “ACL avulsion”)

AND

(“pediatric” OR “children” OR “adolescent” OR “skeletally immature”)

AND

(“anterior cruciate ligament”)

AND

(“management” OR “treatment” OR “surgical fixation” OR “nonoperative management” OR “rehabilitation” OR “return to sport”)

Searches were performed using combinations of free-text keywords and Medical Subject Headings (MeSH) where applicable. Boolean operators (AND/OR) were applied to refine results. No strict filters were applied for study design to allow inclusion of a broad range of clinical and biomechanical evidence.
